# Supplementary material for: The Mean of Milk: A Review of Human Milk Oligosaccharide Concentrations throughout Lactation
Source: Nutrients. 2021 Aug 9;13(8):2737. doi: 10.3390/nu13082737 (PMC8398195; doi:10.3390/nu13082737)
Supplement: Supplementary file 1 [file nutrients-13-02737-s001.zip › nutrients-1280527 supplementary Table S1 - Pooling calculations.pdf]

Review

The Mean of Milk: A Review of Human Milk Oligosaccharide Concentrations throughout Lactation

Buket Soyyilmaz [1,*], Marta Hanna Mikš [1,2], Christoph Hermann Röhrig [1], Martin Matwiejuk [1], Agnes Meszaros-Matwiejuk [1] and Louise Kristine Vigsnæs [1,3]

To understand the calculation of data into a pooled scenario, it is important to know that humans can be categorized into 4 distinct milk groups observed in various frequencies across populations. The milk groups are categorized according to the presence (or absence) of the specific oligosaccharide products of 2 distinct fucosyltransferase enzymes, namely α1,2- and α1,4 fucosyltransferase, encoded by the genes FUT2 and FUT3, respectively.

Milk group 1 is known as Lewis-positive secretor mothers with Lewis blood group (Lea- b+) expressing both FUT2 and FUT3 thus can synthesize both α1,2- and α1,4-fucosylated HMOs. Milk group 2 is Lewis-positive non-secretor mothers with Lewis blood group (Lea+ b-) expressing FUT3 but not FUT2 and thus produce α1,4-fucosylated HMOs but not α1,2-fucosylated HMOs. Milk group 3 is Lewis-negative secretor mothers with Lewis blood group (Le a- b-) expressing FUT2 but not FUT3 and therefore can synthesize α1,2-fucosylated but not α1,4-fucosylated HMOs. Finally, milk group 4 is Lewis-negative non-secretor mothers with Lewis blood group (Lea- b-) expressing neither FUT2 nor FUT 3 therefore cannot produce neither α1,2- not α1,4-fucosylated HMOs.

Table below illustrates the typically observed proportions of the four milk groups in humans. Some studies expressed concentration data simply as secretor (group 1+3) or non-secretor (2+4) while some studies distinctively categorized subjects by one of the four milk groups.

Table S1. Genetic background in population was used as the basis of the pooling calculations, where unpooled levels were converted into a pooled level using the below stated percentage ratios.

Citation: Soyyılmaz, B.; Mikš, M.H.; Röhrig, C.H.; Matwiejuk, M.; Meszaros-Matwiejuk, A.; Vigsnæs, L.K. The Mean of Milk: A Review of Human Milk Oligosaccharide Concentrations throughout Lactation. *Nutrients* **2021**, *13*, 2737. https://doi.org/10.3390/nu13082737

Academic Editor: Hans Demmelmair

Received: 15 June 2021
Accepted: 22 July 2021
Published: 9 August 2021

Publisher's Note: MDPI stays neutral with regard to jurisdictional claims in published maps and institutional affiliations.

Copyright: © 2021 by the authors. Licensee MDPI, Basel, Switzerland. This article is an open access article distributed under the terms and conditions of the Creative Commons Attribution (CC BY) license (http://creativecommons.org/licenses/by/4.0/).

**Secretor Status**	Secretor	Non-secretor	Secretor	Non-secretor
**Milk Group**	1	2	3	4

Milk Phenotype	Se+ / Le (a-b+)	Se- / Le (a+b-)	Se+ / Le (a-b-)	Se- / Le (a-b-)
**α1,2-fucosylated HMOs** (FUT2 enzyme)	+	-	+	-
**α1,3-fucosylated HMOs** (FUT3, FUT5, FUT6 enzymes)	+	+	+	+
**α1,4-fucosylated HMOs** (FUT3 enzyme)	+	+	-	-
**Typically observed frequency**	~ %70	~ %20	~ %9	~ %1
**HMOs contained**	2FL, LNFP I, LNDFH I, LNT, 3FL, DSLNT, LDFT, 6SL, LNFP III, DF-LNH I, LNFP II, LSTc, F-LNH II, LNnT, LNFP V, LNDFH II, 3SL, LSTa, F-LNH I, LNnH, LNH, LSTb, FSL	LNT, 3FL, DSLNT, 6SL, LNFP III, LNFP II, LSTc, F-LNH II, LNnT, LNFP V, LNDFH II, 3SL, LSTa, LNnH, LNH, LSTb, FSL	2FL, LNFP I, LNT, 3FL, DSLNT, LDFT, 6SL, LNFP III, DF-LNH I, LSTc, F-LNH II, LNnT, LNFP V, 3SL, LSTa, F-LNH I, LNnH, LNH, LSTb, FSL	LNT, 3FL, DSLNT, 6SL, LNFP III, LSTc, F-LNH II, LNnT, LNFP V, 3SL, , LSTa, LNnH, LNH, LSTb, FSL

1. When Lewis milk group was not given, secretor (Lewis group 1+3) values were multiplied by 0.8 (80% of the population), and non-secretor (Lewis group 2+4) values were multiplied by 0.2 (20% of the population). This calculation was followed only for the FUT2 dependent HMOs: 2'-FL, LNFP I, LNDFH I, LDFT, DF-LNH I (a), F-LNH I, FS-LNH, S-LNFP I, TF-LNH, DF-LNH III (c).

**Paper**	**Sample**	**Country**	**Lactation phase**	**Milk group**	**Milk type**	**2'-FL**
Sprenger et al. PlosOne 2017	34	Singapore	10 - 60 days	1+3	Secretor	2.170
Sprenger et al. PlosOne 2017	34	Singapore	10 - 60 days	2+4	Non-secretor	0.026

Equation 1)

$2'$-FL$_{1+3}$= 2.180 g/L

$2'$-FL$_{2+4}$= 0.026 g/L

$2'$-FL$_{pooled}$= (2.170 · 0.8) g/L + (0.026 · 0.2) g/L

$2'$-FL$_{pooled}$= 1.7412 g/L

2. When Lewis milk group was specifically given, values were multiplied by 0.7 for milk group 1 (70% of the population), 0.2 for milk group 2 (20% of the population), 0.09 for milk group 3 (9% of the population), 0.01 for milk group 4 (1% of the population). This calculation was followed for all analyzed HMOs.

Paper	Sample	Country	Lactation phase	Milk group	Milk type	3 FL
Austin et al. Nutrients 2019	28	Switzerland	0-5 days	1	Secretor	0,212
Austin et al. Nutrients 2019	28	Switzerland	0-5 days	2	Non-secretor	0,995
Austin et al. Nutrients 2019	28	Switzerland	0-5 days	3	Secretor	0,039
Austin et al. Nutrients 2019	28	Switzerland	0-5 days	4	Non-secretor	0,224

Equation 2)

3FL$_{1}$*= 0.212 g/L*

3FL$_{2}$*= 0.995 g/L*

$$\mathbf{3FL}_3 = 0.039\ g/L$$

$$\mathbf{3FL}_4 = 0.224\ g/L$$

$$\mathbf{3FL}_{pooled} = (0.212 \cdot 0.7)\ g/L + (0.995 \cdot 0.19)\ g/L + (0.039 \cdot 0.1)\ g/L + (0.224 \cdot 0.01)\ g/L$$

$$\mathbf{3FLpooled} = 0.344\ g/L \qquad (2)$$

Numbers in red are the result of pooling calculations. This method of pooling was followed for the 15 papers where trial subjects were divided according to their milk groups.
